# Sequencing and analysis of the complete mitochondrial genome of *Gymnogobius laevis*

**DOI:** 10.1080/23802359.2022.2076625

**Published:** 2022-05-26

**Authors:** Lina Peng, Peilun Li, Hui Li, Wenyan Zhao, Hongkun Wang, Zheng Li, Lili Sun

**Affiliations:** a College of Sport Human Sciences, Harbin Sport University, Harbin, China; b Heilongjiang River Fisheries Research Institute, Chinese Academy of Fishery Sciences, Harbin, China

**Keywords:** *Gymnogobius laevis*, complete mitochondrial genome, phylogenetic analysis

## Abstract

*Gymnogobius laevis* (Steindachner, 1879) is a small, benthic freshwater fish. In a previous study, it was reported as a rare resident in Heilongjiang Province. In this study, the mitochondrial genome of *G. laevis* was sequenced. It is 16,519 base pairs (bp) in length and contains 13 protein-coding genes, two ribosomal RNA genes (12S rRNA and 16S rRNA), 22 transfer RNA genes (tRNA), and two non-coding control regions. The A + T content is 55.71% in the *G. laevis* mitochondrial genome. A phylogenetic tree shows that *G. laevis* is closely related to the family Gobiinae based on complete mitogenome sequences.

*Gymnogobius laevis* (Steindachner, 1879) is a small, benthic freshwater fish that belongs to the family Gobiinae of Perciformes. Gobiinae is one of the largest families in vertebrates (Nelson 2006). Most Gobiinae species like to live in warm waters, and several species live in freshwater rivers. *G. laevis* has been rarely found in Heilongjiang Province. In this study, *G. laevis* specimens were collected from the downstream of Tangwang River (E129°43′38.82″; N46°41′13.92″) in Heilongjiang Province, and its mitochondrial genome was sequenced and analyzed. This study of *G. laevis* has further enriched the fish resources that could provide basic information for other related studies.

All animal experiments were conducted in accordance with the guidelines and approval of the Animal Research and Ethics Committees of Harbin Sport University (approval number HTY20200711). The fins of *G. laevis* were collected for DNA extraction and the sample was stored in the herbarium of the Heilongjiang River Fisheries Research Institute (accession number: HRFRI-S-2021020; specimen ID: https://www.hrfri.ac.cn/; Li Peilun; lipeilun@hrfri.ac.cn). DNA was collected according to the cetyltrimethylammonium bromide (CTAB) DNA extraction protocol (Zhai et al. 2021). The complementary DNA (cDNA) library was constructed and sequenced by using the Illumina NovaSeq at Jin Bei Biotech (Harbin, China). Approximately, 4.5 Gb of raw data were obtained, and the clean reads (29,720,032) were spliced using the SPAdes software (version 3.13) (Bankevich et al. 2012). The mitochondrial genome of *G. laevis* was annotated using MITOS (http://mitos.bioinf.uni-leipzig.de; Bernt et al. 2013). The mitochondrial sequence has been deposited in GenBank under the accession number MW049117.

The mitochondrial DNA sequence is 16,519 bp in length and contains 13 protein-coding genes, two ribosomal RNA genes (12S rRNA and 16S rRNA), 22 transfer RNA genes (tRNA), and two non-coding control regions. The A + T content is 55.71%. The 13 protein-coding genes are encoded on the light strand, except for *ND6*; these genes vary from 297 to 1554 bp in length. The genes *ND1*, *ND2*, *COX2*, *ATP9*, *ATP6*, *COX3*, *ND3*, *ND4L*, *ND4*, *ND5*, *ND6*, and *CYTB* begin with ATG as the start codon, while *COX3* begins with GTG. The 22 tRNA genes are from 66 bp (tRNA-Phe) to 75 bp (tRNA-Lys) in length; they present a typical secondary structure. The two rRNA genes are 949 and 1637 bp. The 12S rRNA gene is located between tRNA-Phe and tRNA-Val, and the 16S rRNA gene is located between tRNA-Val and tRNA-Leu. The two non-coding control regions are 31 and 508 bp.

The mitochondrial DNA was submitted to multiple alignments by using Clustal W 2.0. The phylogenetic tree was constructed by using the maximum-likelihood method under the Tamura-Nei model, with 1000 bootstrap replicates in the MEGA 6.0 software based on the nucleotide sequences (Tamura et al. 2013). The mitochondrial genome sequences are used from 10 species in the Gobiinae family and three other families – Odontobutidae, including *Micropercops swinhonis* (Gunther, 1873) and *Perccottus glenii* (Dybowski, 1877); Siluriformes, including *Clarias fuscus* (Lacepede, 1803); and Cyprinidae, including *Mylopharyngodon piceus* (Richardson, 1846), which was used as an outgroup (Mascolo et al. 2018). *G. laevis* clusters with other members of the family Gobiinae and diverges from the other three families (Figure 1). We hope that the present results help to clarify the taxonomic status of *G. laevis*: it is closely related to the family Gobiinae based on the mitochondrial genome.

**Figure 1.**
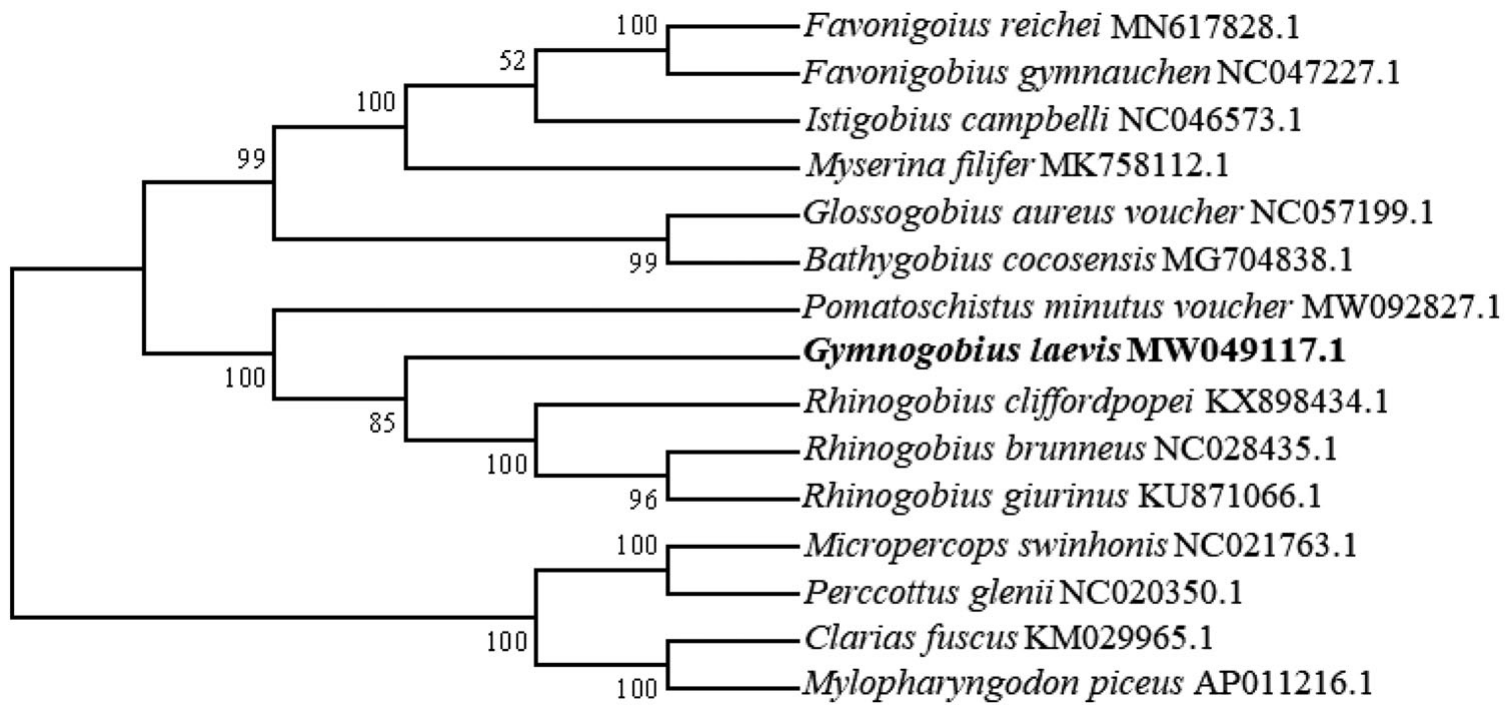
Molecular phylogenetic analysis of *Gymnogobius laevis*. The phylogenetic tree was prepared by using the maximum-likelihood method under Tamura-Nei model with 1000 bootstrap replicates in the MEGA 6.0 software.

## Data Availability

The genome sequence data that support the findings of this study are openly available in GenBank of NCBI (http://www.ncbi.nlm.nih.gov/) under accession no. MW049117.1. The associated BioProject, SRA, and Bio-Sample numbers are PRJNA802364, SRR17659578, and SAMN 25538277, respectively.
